# Effects of image-sharpening algorithm on surgical field visibility during 3D heads-up surgery for vitreoretinal diseases

**DOI:** 10.1038/s41598-023-29882-5

**Published:** 2023-02-16

**Authors:** Kosuke Nakajima, Makoto Inoue, Masaharu Mizuno, Takashi Koto, Tomoka Ishida, Hitomi Ozawa, Tetsuro Oshika

**Affiliations:** 1 Kyorin Eye Center, Kyorin University School of Medicine, 6-20-2 Shinkawa, Mitaka, Tokyo, 181-8611 Japan; 2 Department of Clinical Engineering, Kyorin University Hospital, 6-20-2 Shinkawa, Mitaka, Tokyo, 181-8611 Japan; 3 Department of Ophthalmology, Faculty of Medicine, University of Tsukuba, 1-1-1 Tennoudai, Tsukuba, 305-8575 Japan

**Keywords:** Retinal diseases, Translational research

## Abstract

We conducted clinical and experimental studies to investigate the effects of image-sharpening algorithms and color adjustments, which enabled real-time processing of live surgical images with a delay of 0.004 s. The images were processed with image-sharpening intensities of 0%, 12.5%, 25%, and 50% during cataract surgery, vitrectomy, peeling of epiretinal membrane, and peeling of internal limiting membrane (ILM) with the Ngenuity 3D visualization system. In addition, the images obtained with a yellow filter during the ILM peeling were processed with color adjustments. Five vitreoretinal surgeons scored the clarity of the images on a 10-point scale. The images of a 1951 United States Air Force grating target placed in no fluid (control), saline, and 0.1% and 1% milk solution were evaluated. The results showed that the mean visibility score increased significantly from 5.0 ± 0.6 at 0% to 6.4 ± 0.6 at 12.5%, 7.3 ± 0.7 at 25%, and 7.5 ± 0.9 at 50% (*P* < 0.001). The visibility scores during ILM peeling improved significantly with color adjustments (*P* = 0.005). In the experimental study, the contrast of the grating targets blurred by the 0.1% and 1% milk solution increased significantly by the image-sharpening procedure. We conclude that the image-sharpening algorithms and color adjustments improved the intraoperative visibility of 3D heads-up surgery.

## Introduction

During heads-up surgery, the surgical field observed through an operating microscope is displayed on a 55-inch 3D monitor, and this technique has been used in ophthalmic surgery^1–12^. This system allows the surgeon to observe an enlarged 3D image of the surgical field^8–10^. The ability to perform vitreoretinal surgery successfully under low lighting conditions is due to the improved sensitivity of the video cameras, and surgery under low light conditions is helpful because it reduces retinal phototoxicity^13–16^. Comparisons of 3D head-up surgery to surgery with conventional surgical microscopes have shown that the outcomes of heads-up surgery were equal or even superior to conventional surgery^17–26^. It has a better depth of focus but there is less lateral resolution^5,6^. Heads-up surgery has been shown to be suitable for beginners^27,28^, and it is better for the ergonomics of the surgeons^13,22^.

However, surgical images with rapid changes in brightness are affected by the limitations of the digital equipments^9^. These digital factors are the number of pixels, the dynamic range of the digital cameras, the number of pixels of the 3D monitors, and the transfer rate and processing speed of the heads-up surgical equipment^2^. The digital images of heads-up surgery have characteristics that are different from the actual surgical images observed through the operating microscope^9^. On the other hand, digitized images have the potential of facilitating surgical operations by altering different digital processes. For example, adjusting the brightness and contrast by intraocular dyes such as Brilliant blue G (BBG) to peel the internal limiting membrane (ILM), and color adjustments using color channels that enhance the intensity of the dye which can make it easier to detect and peel the ILM^3^.

Image-sharpening technology and deep learning algorithms have been used in radiographic and magnetic resonance imaging^29–32^ and also in the ophthalmology field^33–35^. Thus, Hoshi and associates^33^ reported that image processing with honeycomb-removal and image-sharpening algorithms improved the dacryoendoscopic visibility. Tasaki and associates^34^ reported that image processing with honeycomb-removal and image-sharpening algorithms significantly improved the visibility of the surgical field in 27-gauge endoscopic vitrectomy. Akiyama and associates^35^ reported that the clarity of the digital image enhancement during heads-up surgery improved the surgical outcomes. However, to the best of our knowledge, there has not been a comprehensive study on the use of image-sharpening algorithms in improving the clarity of the surgical field during 3D heads-up ophthalmologic surgery.

Thus, the purpose of this study was to determine whether image-sharpening algorithms can improve the clarity of the surgical field during heads-up surgery. We also examined whether color enhancement algorithms can improve the visibility of the surgical field during heads-up surgery.

## Material and methods

### Clinical study approval

This was a single-center, prospective clinical study, and the procedures were approved by the Institutional Review Committee of the Kyorin University School of Medicine (1904). The procedures adhered to the tenets of the Declaration of Helsinki. All of the patients received a detailed explanation of the surgical and ophthalmic procedures including the use of the medical image enhancer, and all signed an informed consent form. All of the patients consented to our review of their medical images and their anonymized use in medical publications.

### Surgical procedures

We performed 27-gauge combined pars plana vitrectomy (PPV) and cataract surgery with implantation of an intraocular lens (IOL) on 4 eyes of 4 patients. All of the surgical procedures were performed under local anesthesia by a single surgeon (MI). In addition to the cataract surgery, the surgeries were performed on an eye with epiretinal membrane, an eye with epiretinal membrane associated with a peripheral retinal detachment, on an eye with vitreomacular traction syndrome, and on an eye with vitreomacular traction syndrome associated with vasoproliferatiive tumor.

The 3D heads-up surgery was performed with the Ngenuity 3D Visualization System (ver. 1.4, Alcon Laboratories, Fort Worth, TX) and the Medical Image Enhancer (MIEr, Logic & Design, Tokyo, Japan) with an image-sharpening intensity of 12.5%. The Rescan 700 operating microscope (Carl Zeiss Meditec, Oberkochen, Germany) and the Constellation Vision System (Alcon Laboratories, Fort Worth, TX) were used for the 27-gauge PPV. The cataractous lens was removed by phacoemulsification, and the residual posterior hyaloid cortex was made more visible by intravitreal injection of triamcinolone acetonide (MaQaid, Wakamoto Pharmaceutical Co., LTD, Tokyo, Japan). Any residual posterior hyaloid cortex was removed by suction with a vitreous cutter. The epiretinal membrane and the ILM were peeled in all eyes. For the ILM peeling, BBG was used to increase the visibility of the ILM with a yellow color channel setting (Cyan Red; 80, Magenta Green; 100, Yellow Blue; 66, Brightness; 47.52, Contrast; 58.00, Gamma; 1.2, Hue; 17, Saturation; 63) on the Ngenuity system. All cases underwent cataract surgery and implantation of an IOL in the capsular bag before the PPV. Endo-photocoagulation and tamponade by air or 20% sulfur hexafluoride (SF6) were performed as needed. A video of the surgery was recorded with the Ngenuity 3D Visualization System in the original condition without image-sharpening. The video images were extracted under different intensities of image-sharpening by the medical image enhancer.

Five surgical images were extracted for each surgical procedure: anterior lens capsulotomy with continuous curvilinear capsulorhexis (CCC), phacoemulsification and aspiration (PEA), cortical aspiration with irrigation and aspiration (I/A), vitrectomy (Vit) with peeling of an epiretinal membrane (ERM), and peeling of the ILM. The 30 original surgical images were processed at four levels of sharpening intensities of 0% (original; no image-sharpening), 12.5%, 25%, and 50%. In addition, 10 images of ILM peeling after intravitreal injection of BBG and the yellow color channel were processed with original (0%; no sharpening and color adjustments), image-sharpening intensity of 25% and no color adjustment, image-sharpening intensity of 25% and color adjustment of 150%, image-sharpening intensity of 25% and color adjustment of 200% were processed.

Five vitreoretinal surgeons (KN, MM, TI, TK, MI) of intermediate or higher surgical experiences were the evaluators, and each of the 40 surgical images representing each procedure with less blurring was scored on a 10-point scale for each of the three conditions based on the following points with masked medical data and the use of the image sharpening algorithms other than the images. (1) More than image clarity, surgery can be performed safely and accurately, (2) the intraoperative manipulation site is clearer, and (3) the background is not too emphasized but the intraoperative manipulated site is. The scores of the original and processed images were statistically compared.

### In vitro studies of image-sharpening

**Figure 1:**
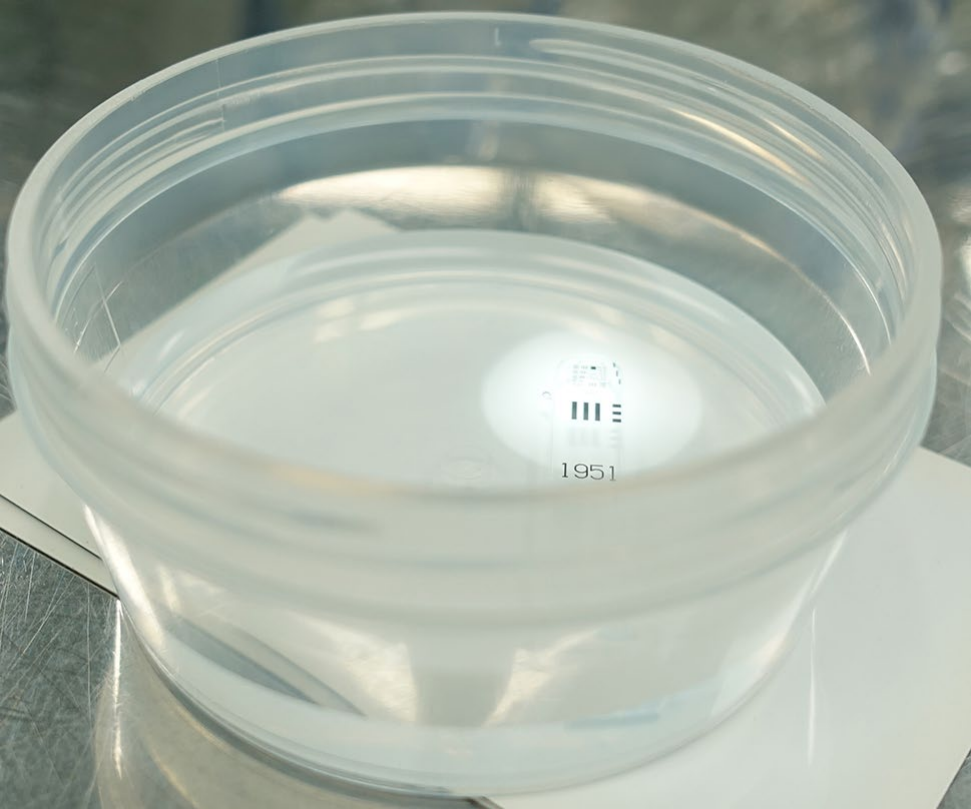
Schema of the in vitro analysis. A transparent 4 cm diameter laboratory dish is placed on white paper, and a 1951 United States Air Force (USAF) test grating target printed on the glass plate is placed on the bottom of the dish. The coaxial light source with 0-degree illumination of the operating microscope was used to illuminate the grating targets.

In vitro studies were performed to evaluate the effectiveness of the image-sharpening algorithms. A transparent laboratory dish of 4 cm diameter was placed on white paper, and a 1951 United States Air Force (USAF) test target grating (Edmund Optics, Barrington, NJ) printed on a glass plate was placed on the bottom of the dish (Fig. 1). Images of the dish without fluid (control), or filled with 5 ml of saline, or 0.1% or 1% milk solution (MS) diluted with saline. The coaxial light source of the operating microscope was used for 0-degree illumination. The images of the grating of the USAF charts were recorded with the Ngenuity 3D Visualization System (Alcon Laboratories, Fort Worth, TX) and the medical image enhancer (MIEr, Logic & Design, Tokyo, Japan) through the RESCAN 700 (Carl Zeiss Meditec, Oberkochen, Germany) surgical microscope with a maximum magnification of 25.5 X. To evaluate the differences in the quality of the images quantitatively, static images were produced from the video images of the Ngenuity 3D Visualization System, and the pixel intensity of the dark grating bar and the space between the bars of a 2–3 scale (5.04 cycle/mm) gratings were measured with the blue color information scale of Photoshop CS6 software (Adobe System Inc., CA). The mean intensity at the center of the dark grating bar was set as I_min_ and the intensity at the center of the bright space was set as I_max_. The contrast was calculated as (I_max_ – I_min_) / (I_max_ + I_min_).

### Statistical analyses

The significance of the differences in the quality of the images after the sharpening intensities was compared with the Friedman test. The 2 groups comparisons were determined by the Wilcoxon signed rank tests, Mann–Whitney *U* tests, or Fisher’s exact probability tests. All statistical analyses were performed using SPSS (version 28.0; IBM, Armonk, New York, NY, USA).

### Institutional review board statement

The Institutional Review Committee of the Kyorin University School of Medicine approved the study protocol (1904). This study was conducted in accordance with the Declaration of Helsinki.

### Informed consent statement

Informed consent for data acquisition was obtained from all subjects involved in the study.

## Results

All of the surgeries were performed successfully without any intraoperative complications with the Ngenuity 3D Visualization System and the Medical Image Enhancer with an image-sharpening intensity of 12.5%. During the surgery, none were switched to the 3D heads-up surgery alone without the Medical Image Enhancer. All of the surgeries were completed without any complications.

### Visibility scores of surgical procedures

**Figure 2:**
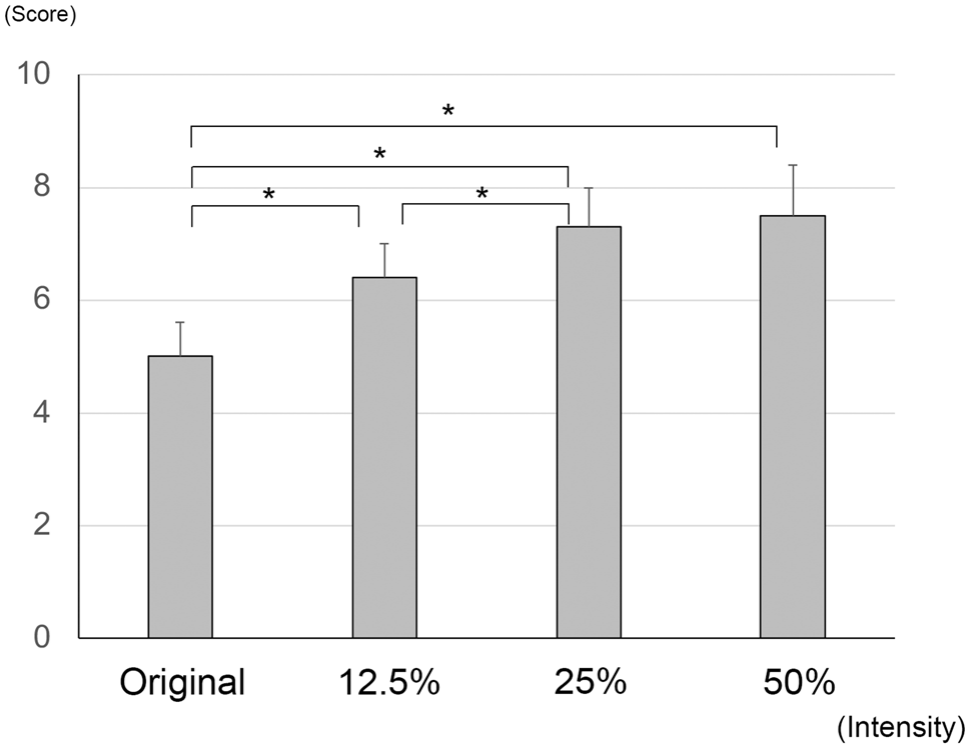
Mean visibility score for all procedures. The mean visibility score increases significantly from 5.0 ± 0.6 at 0% (original source) to 6.4 ± 0.6 at 12.5% intensity of the image-sharpening (*P* < 0.001, Wilcoxon signed rank tests) and 7.3 ± 0.7 at 25% (*P* < 0.001). The mean visibility score increases to 7.5 ± 0.9 at 50%, but the increase from the original source is not significant (*P* = 0.294). *; *P* < 0.001.

The mean visibility score increased significantly from 5.0 ± 0.6 at 0% (original source) to 6.4 ± 0.6 at 12.5% (*P* < 0.001, Wilcoxon signed rank tests; *P* < 0.012 was taken to be significant with Bonferroni correction) and to 7.3 ± 0.7 at 25% (*P* < 0.001). The mean visibility score increased to 7.5 ± 0.9 at 50%, but the increase from the original source was not significant (*P* = 0.294, Fig. 2). The visibility scores of CCC, PEA, I/A, Vit, ERM, and ILM were significant for 0%, 12.5%, 25%, and 50% intensities of the image-sharpening algorithms (Table 1). The visibility scores of CCC, PEA, I/A, Vit, ERM, and ILM increased significantly from the original source (0%) to 12.5% intensity of the image-sharpening (n = 5, CCC, *P* = 0.041; PEA, *P* = 0.039; I/A, *P* = 0.043; Vit, *P* = 0.042; ERM, *P* = 0.042; ILM, *P* = 0.042). The visibility scores of the ILM increased significantly with color adjustments from 100 to 150% (n = 10, *P* = 0.005).

**Table 1:** Visibility scores of surgical images in ease of performing surgery by the vitreoretinal surgeons.

Conditions	All	CCC	PEA	I/A	Vit	ERM	ILM	Conditions	Color
Original (0%)	5.0 ± 0.6	4.3 ± 0.3	4.8 ± 0.4	5.6 ± 0.4	5.6 ± 0.4	4.8 ± 0.1	5.0 ± 0.2	Source (0%)	4.6 ± 0.4
12.5%	6.4 ± 0.6	5.6 ± 0.3	6.0 ± 0.3	7.0 ± 0.4	6.5 ± 0.6	6.6 ± 0.4	7.1 ± 0.3	100%	5.9 ± 0.6
25%	7.3 ± 0.7	6.8 ± 0.4	7.3 ± 0.5	8.0 ± 0.4	6.6 ± 0.2	7.4 ± 0.7	8.1 ± 0.3	150%	7.2 ± 0.8
50%	7.5 ± 0.9	7.9 ± 1.0	7.4 ± 0.9	7.9 ± 0.5	6.1 ± 0.6	7.5 ± 0.5	8.2 ± 0.4	200%	7.4 ± 1.2
Friedmann test*	< 0.001	0.009	0.004	0.003	0.009	0.003	0.003	Friedmann test*	< 0.001
*P*-value**	< 0.001	0.041	0.039	0.043	0.042	0.042	0.042	*P*-value***	0.005

CCC, continuous circular capsulorhexis; PEA, phacoemulcification and aspiration; I/A, irrigation and aspiration; Vit, vitrectomy; ERM, epiretinal membrane peeling; ILM, internal limiting membrane peeling; Color, color adjustments.

*Friedmann test among 4 conditions, **Wilcoxon sign-rank test between the original and 12.5% intensity, ***Wilcoxon sign-rank test between 100 and 150% of color adjustments.

**Figure 3:**
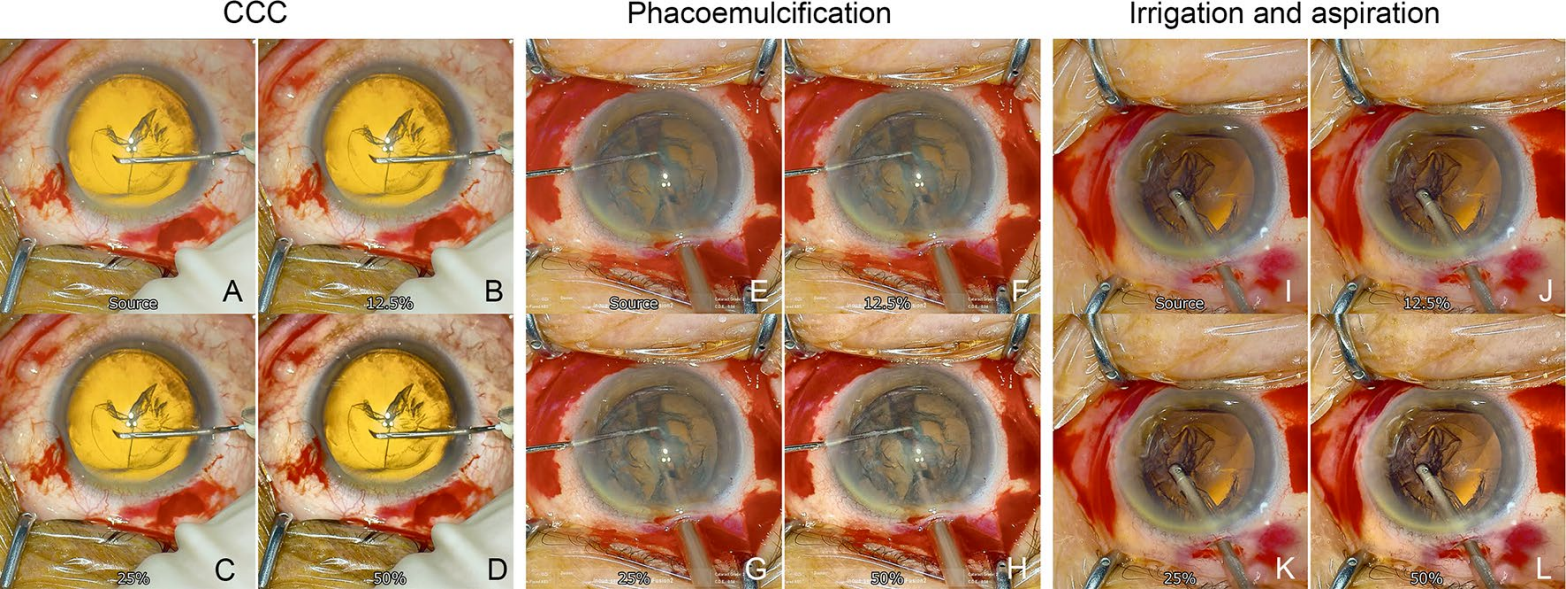
Surgical images during cataract surgery. Surgical image during anterior capsulotomy by continuous curvilinear capsulorhexis (CCC) with the original image (**A**), 12.5% (**B**), 25% (**C**), and 50% (**D**) intensity of image-sharpening algorithms. The images show that the edge of the anterior capsulotomy is sharper with increasing intensity of the image-sharpening algorithms. In addition, the patterns of the conjunctival vessels are also enhanced with the increase of the intensity of the image-sharpening. Surgical images during phacoemulsification of the lens nucleus with the original image (**E**), 12.5% (**F**), 25% (**G**), and 50% (**H**) intensity of image-sharpening indicate that the edge of the lens nucleus appears to be sharper with increasing intensity of the image-sharpening algorithms. Surgical images during aspiration of the lens cortex with the original image (**I**), 12.5% (**J**), 25% (**K**), and 50% (**L**) intensity of image-sharpening show that the edge of the lens cortex appears to be sharper with increasing intensity of the image-sharpening.

For CCC, the edge of the anterior lens capsule was clearer with increasing intensity of the image-sharpening procedure (Fig. 3). In contrast, the visibility of the conjunctival vessels was also enhanced with the increase of the intensity of the image-sharpening algorithms. In PEA, the edge of the lens nucleus appeared clearer with an increase in the intensity of the image-sharpening algorithms. The bottom of the lens nucleus also appeared clearer with image-sharpening indicating that the depth of focus was increased. In the I/A procedure, the edge of the anterior capsule and lens cortex appeared clearer with an increase in the intensity of the image-sharpening algorithms.

**Figure 4:**
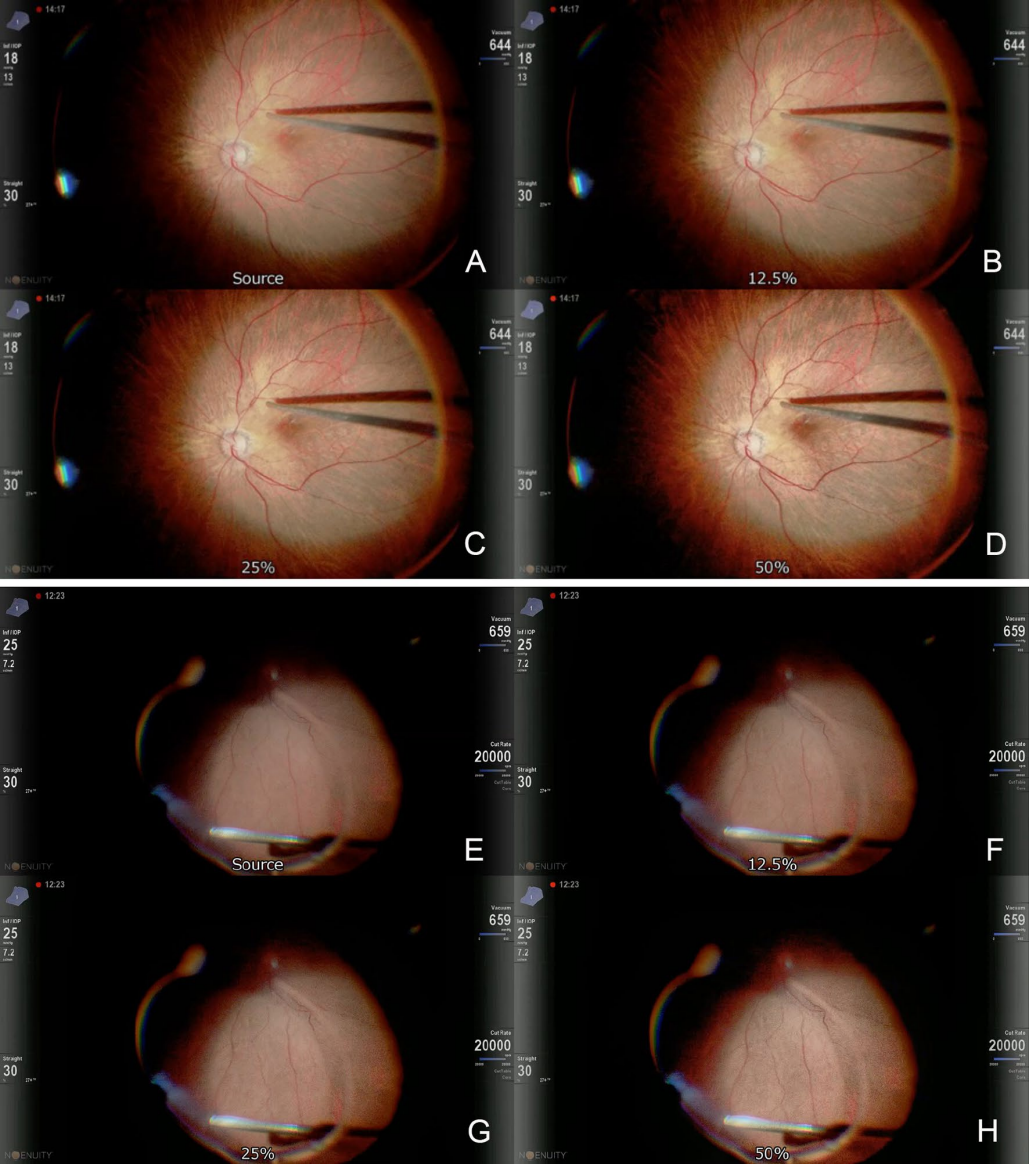
Surgical images during core vitrectomy. Surgical images during core vitrectomy under a wide-angle viewing system with the original image (**A**), 12.5% (**B**), 25% (**C**), and 50% (**D**) intensity of image-sharpening showing that the retinal image and the pattern of the retinal vessel and choroidal vessels are more clearly delineated in the shadow of the intraocular illumination with the increase of the image-sharpening. Surgical images during peripheral vitrectomy under a wide-angle viewing system with the original image (**E**), 12.5% (**F**), 25% (**G**), and 50% (**H**) intensity of image-sharpening indicate that the vitreous fibers illuminated by the intraocular illumination are more clearly depicted with the increase of the image-sharpening algorithms.

In the Vit procedure, the shape of the vitreous cutter and the pattern of the retinal and choroidal vessels appeared clearer with increases in the intensity of the image-sharpening procedure (Fig. 4). The retinal image was also more clearly delineated in the shadow of the intraocular illumination. Vitreous fibers illuminated by the intraocular illumination were also seen more clearly and they were observed as white fibers in front of the retina, The fibers moved forward and backward by the flow of the fluid from the intraocular infusion and the aspirating flow by the vitreous cutter. However, the stable images were not as different as those seen in the video images.

**Figure 5:**
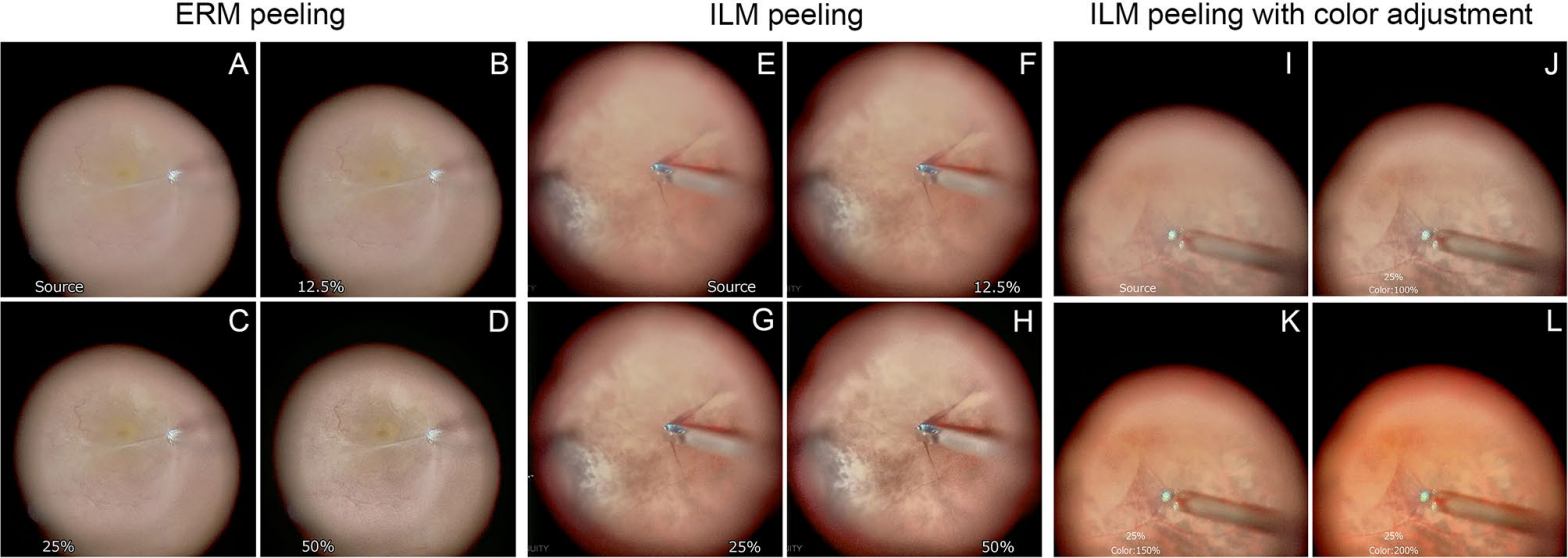
Images during the peeling of an epiretinal membrane (ERM) and an internal limiting membrane (ILM). Surgical images during ERM peeling seen through a flat contact lens with the original image (**A**), 12.5% (**B**), 25% (**C**), and 50% (**D**) intensity of image-sharpening show that the edge of ERM appears clearer with the image-sharpening and the patterns of retinal vessels and retinal folds also appear more clearly with the increase of the image-sharpening intensity. The images during ILM peeling with the aid of the yellow filter through a flat contact lens with the original image (**E**), and with 12.5% (**F**), 25% (**G**), and 50% (**H**) intensities of the image-sharpening showing that the edge of ILM stained blue with BBG appears clearer with the image-sharpening. Images of the surgical field during ILM peeling with color adjustments by a yellow filter with the original image (**I**), 12.5% of the image-sharpening intensity (**J**), 150% of color adjustment and 12.5% of the image-sharpening intensity (**K**), and 200% of color adjustment and 12.5% of the image-sharpening intensity (**L**) indicate that the peeled ILM appears clearer with the color adjustment. The yellow color is emphasized more than enhanced by the yellow filter, and the color tone is observed more clearly with the color adjustment. In particular, the BBG-stained ILM and unstained areas are more clearly delineated.

During the peeling of the ERM, the edge of the ERM appeared clearer with image-sharpening, and the patterns of the retinal vessels and retinal folds also appeared clearer with an increase in the image-sharpening intensity (Fig. 5). In ILM peeling with the yellow filter, the edge of the ILM stained blue with BBG and appeared clearer with image-sharpening. In ILM peeling with color adjustments and the yellow filter, the ILM appeared more clearly with the color adjustments, and the yellow color was emphasized more than that enhanced by the yellow filter. The color was observed more clearly with the color adjustments. In particular, the BBG-stained ILM and unstained areas were delineated more clearly.

### In vitro analyses of the effectiveness of image-sharpening algorithms

**Figure 6:**
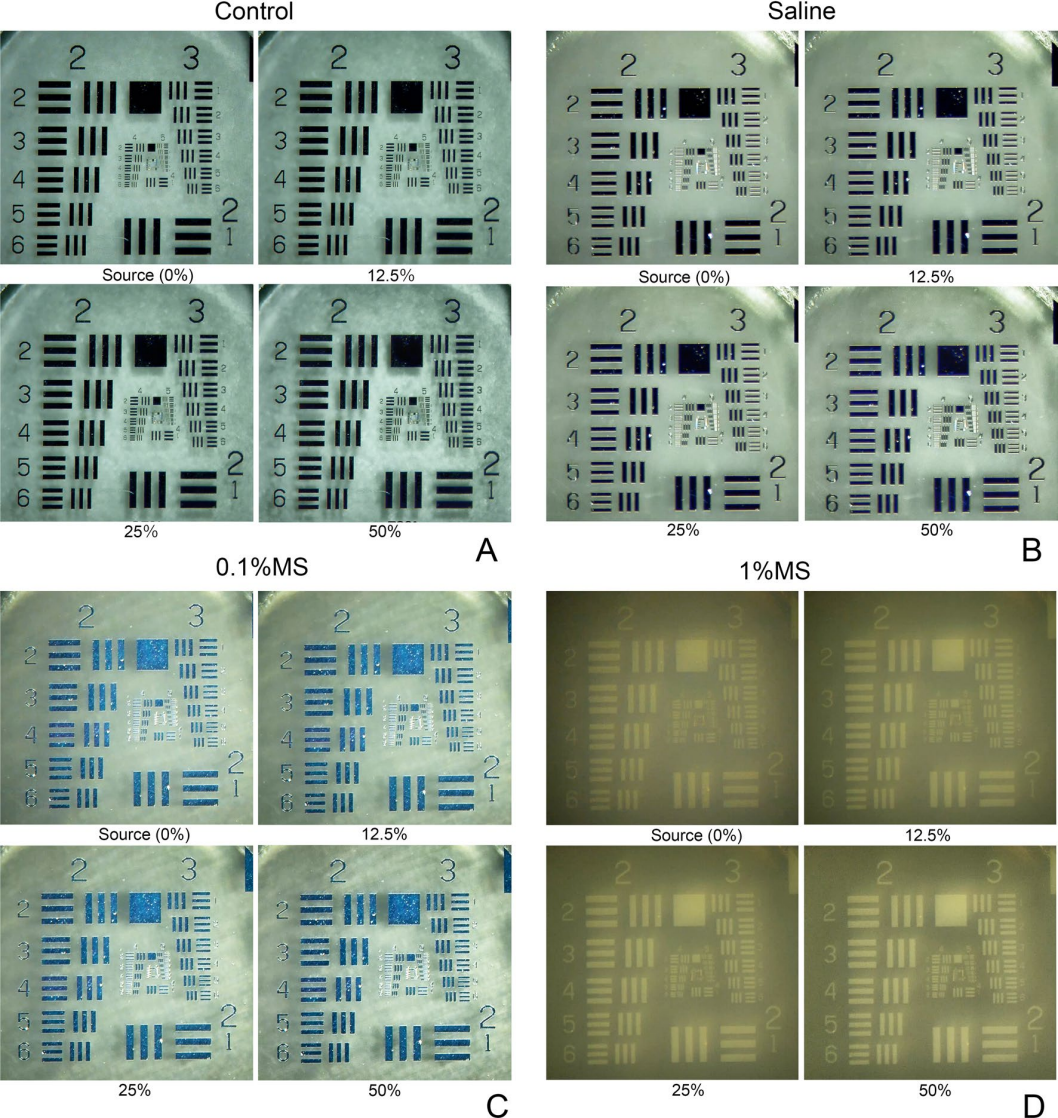
Images of the 1951 United States Air Force (USAF) grating targets through saline and milk solution (MS). (**A**) In the control (no fluid), the grating images are not enhanced when the image-sharpening intensity is increased, but its shadows are enhanced and the white background behind the targets appears brighter. Through the saline solution (**B**), the grating target images are only slightly enhanced even when the image-sharpening intensity is increased, but the enhancement of the shadows of the grating target images seen in the control is reduced. The white background behind the targets appears brighter with an increase in the image-sharpening intensity which was also less than the increase of the control. At 0.1% MS (**C**), the entire index images were bluish-green in color. The grating targets are more contrast-enhanced when the image-sharpening intensity is increased. The shadows of the grating targets and the white background appear more enhanced and the background speckle pattern is emphasized more with increasing image sharpness compared to saline. At 1% MS (**D**), the entire index images appear darker probably because light does not reach it. When the image-sharpening intensity is increased, the target images become brighter and the background darker, and they are observed more clearly due to the increased contrast. As the image-sharpening intensity increases, even fine details of the indices that are blurred can be observed, and the resolution is observed to have improved under the 1% MS condition. For example, the 4–5 and 4–6 indicators are indistinguishable without image-sharpening but becomes clearer as the intensity of image-sharpening is increased to 25% and 50%.

In the control without any fluid in the dish, the black bars of the grating target did not change significantly when the image-sharpening intensity was increased, but its shadows were enhanced and the white background behind the targets appeared brighter (Fig. 6). With the saline solution, the images of the grating were only slightly enhanced even when the image-sharpening intensity was increased, but the enhancement of the shadows of the grating target images seen in the control was reduced more than that of the control. This was probably due to the diffuse reflection of the water. The white background behind the targets appeared brighter with an increase in the image-sharpening intensity which was also less than the increase of the control. With 0.1% MS, the entire image was bluish-green. The contrast of the targets was more enhanced when the image-sharpening intensity was increased. The shadows of the grating and the white background appeared more enhanced, and the speckle pattern of the background was emphasized more with increasing image sharpness compared to that in saline. With 1% MS, the entire image appeared darker probably because the light did not reach it. The images of the grating target appeared to be whitish due to reflections from the surface of the indicator and the white background was much darker. These changes resulted in an inversion of the black-and-white areas under these conditions. When the image-sharpening intensity was increased, the images of the target became brighter and the background darker, and they were observed more clearly due to the increase in contrast. As the image-sharpening intensity increased, even the fine details of the indices that were blurred could be observed, and the resolution improved under the 1% MS condition. Thus, the 4–5 and 4–6 indicators were indistinguishable without image-sharpening but became clearer as the intensity of image-sharpening was increased to 25% and 50% (Fig. 6).

**Figure 7:**
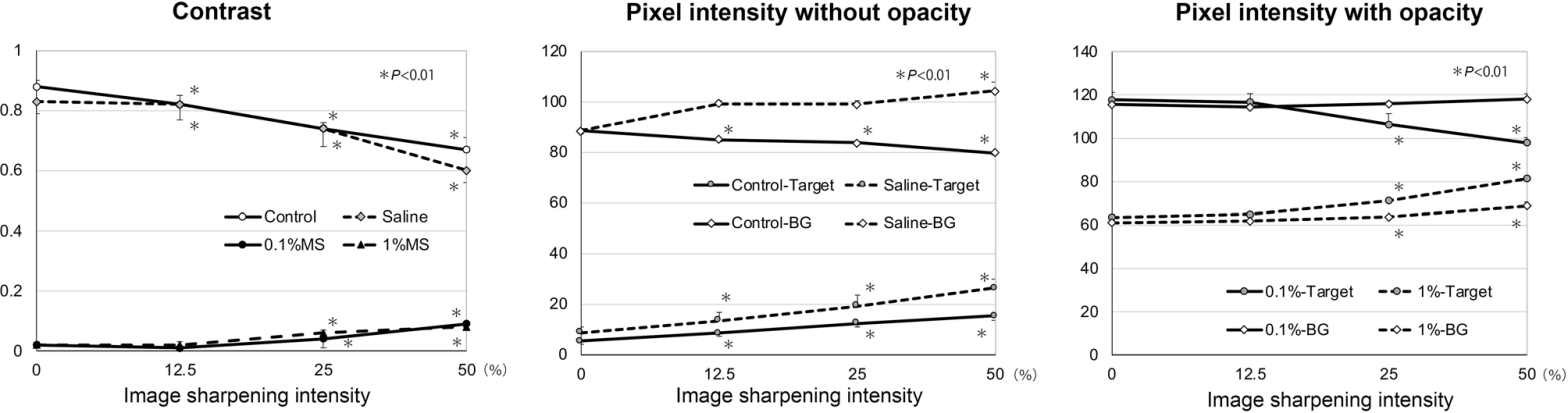
The contrast and the pixel intensity of the images of the grating target. The contrast decreases significantly with an increase of image-sharpening intensity in the control (no fluid) and in saline. On the other hand, the contrast increases significantly with the increase of image-sharpening intensity in 0.1% milk solution (MS) and 1% MS at the intensity of 25% and 50%. The pixel intensity of the grating targets images increases significantly with the increase of image-sharpening intensity in the control and saline. The pixel intensity of the background (BG) between the grating targets images decreases significantly with the increase of image-sharpening intensity in the control. In contrast, the pixel intensity of the BG gradually increases with the increase of image-sharpening intensity in the saline and increases significantly at the image-sharpening intensity of 50%.

The contrast decreased significantly with an increase in the image-sharpening intensity in the control without a solution and also in the saline solution (Fig. 7). In contrast, the contrast increased significantly with an increase in the image-sharpening intensity in 0.1% MS and 1% MS at intensities of 25% and 50%. The pixel intensity of the images of the grating targets increased significantly with an increase in the image-sharpening intensity in the control and in the saline solution. The pixel intensity of the background between the images of the grating targets decreased significantly with an increase in image-sharpening intensity in the control. In contrast, the pixel intensity of the background gradually increased with an increase of the image-sharpening intensity in the saline and increased significantly at an image-sharpening intensity of 50%. The pixel intensity of the images of the grating targets decreased significantly with an increase in the image-sharpening intensity in 0.1% MS but increased in 1% MS. The pixel intensity of the background between the images of the grating targets did not change with an increase in image-sharpening intensity in 0.1% MS but increased significantly in 1% MS. These results showed that the image-sharpening algorithms were not just a process of increasing the contrast uniformly.

## Discussion

Our results showed that the image-sharpening algorithms improved the clarity of all objects in the surgical field during simultaneous cataract and vitreous surgery using a 3D heads-up viewing system. The algorithms were tuned to improve the clarity of live images by increasing the dynamic range per pixel calculation in real-time by 0.004 sec^33,34^. The dynamic range of the surgical images can be expanded by conventional methods such as gain correction, gamma correction, tone curve correction, and histogram flattening^36,37^. These methods can adjust the overall brightness and darkness uniformly but they cannot manage images where the bright and dark areas are mixed in the same image. High Dynamic Range (HDR) video cameras combine multiple images from different exposures to improve the dynamic range balance of bright and dark areas of the same image, but they cannot handle partial clouding or general haze in the image^36,37^.

The proprietary image-sharpening algorithms used in this study enhanced the image quality by optimizing contrast and restoring the resolution of the images by narrowing the point-spread function^33,34^. In addition, an extended dynamic range was attained with an HDR video camera equipped with the Ngenuity 3D Visualization System. The results showed that the image-sharpening algorithms improved the visibility scores significantly in the various surgical procedures during simultaneous cataract and vitreous surgery.

In the image processing that adjusted the dynamic range of the contrast, usually only the brightness information is changed without adjusting the color information^36,37^. Therefore, image processing occasionally has a problem that modifies the images to appear as black and white images with no adequate color adjustments. In this algorithm of the medical image enhancer, the color tone is calculated from the ratio of brightness change for each pixel unit and then processed to create natural colors for the entire image as a parameter of color adjustment by regulating the coefficient at the time of the calculations. Akiyama and associates^35^ evaluated the efficacy of the digital image-processing enhancement by the algorithm of the Advanced Image Multiple Enhancer during heads-up surgery during vitrectomy and ILM peeling assisted with BBG staining. Image processing increased the mean color difference significantly and reduced the light intensities during vitrectomy.

The clarity of the surgical images was not only enhanced in the area where the surgical operation was being performed but also in the surrounding image. The epiretinal membrane and other areas were also enhanced as were the retinal folds, retinal vessels, and choroidal vessels. Thus, the evaluation was made from three perspectives. Although the visibility score was improved by the image-sharpening algorithms and the color adjustments, it was a subjective evaluation by the evaluators, and in some surgical situations, the visibility score decreased when the intensity of image-sharpening was excessively increased. Therefore, the ability to adjust the intensity of image-sharpening as a parameter is an advantage for ophthalmic surgery.

Our in vitro experiments were performed to obtain an objective evaluation of the image-sharpening algorithms. We found that the image-sharpening algorithms adjusted and normalized the contrast for each situation. In the condition without opacity of the control and saline, the contrast decreased as the image-sharpening intensity increased while the image-sharpening algorithms improved the visibility scores of the surgical images. These appear to be different from the results of the surgical images. The surgical images were sharp, in-focus images that had been processed with the image sharpening algorithms, so it was close to the conditions without opacities in the control and saline in the in vitro experiments. Under these conditions of clear media, the image sharpening algorithms attempt to normalize the image by performing pixel-by-pixel calculations on uniform targets and backgrounds which will result in an enhancement of the bright points in dark targets and dark points in bright backgrounds. Consequently, the contrast decreased in the condition without opacities when the image sharpening intensity increased. On the other hand, in the condition with an opacity of 0.1% and 1% MS, the algorithms increased the contrast significantly with an increase in the image-sharpening intensity because the targets and the background were blurred. Thus, the enhancement of each pixel within the target and the background was less likely to be performed and the margins of the targets were more enhanced. We also found that the resolution can be improved when the original visibility was markedly reduced. Although digitally assisted surgery has some limitations in terms of resolution^5,6^, it is expected to become more useful in the future as a digital platform that integrates a large amount of image information and performs digital processing such as image-sharpening^7^.

The study had several limitations. First, although the study design was prospective, the small number of cases did not allow a direct assessment of the improvement in the outcomes with image clarification. Further studies are needed to determine whether the proposed algorithms will contribute to improved surgical outcomes, especially in cases with poor visibility. Second, the image-sharpening device could not be validated in patients with significantly poor intraoperative visibility. Thus, future studies are warranted as the in vitro studies have shown that the device can be expected to be useful in patients with poor visibility.

In conclusion, the image-sharpening algorithms and color adjustments processed live images in real-time and enhanced the intraoperative visibility during 3D heads-up surgery with the Ngenuity 3D Visualization System by not only increasing the contrast but also optimizing contrast and restoring the resolution of the images by narrowing the point-spread function.

## Data Availability

The datasets analyzed and generated during the current study are available from the corresponding author upon reasonable request.
